# Broad-Spectrum Cephalosporin-Resistant *Klebsiella* spp. Isolated from Diseased Horses in Austria

**DOI:** 10.3390/ani10020332

**Published:** 2020-02-20

**Authors:** Igor Loncaric, Adriana Cabal Rosel, Michael P. Szostak, Theresia Licka, Franz Allerberger, Werner Ruppitsch, Joachim Spergser

**Affiliations:** 1 Institute of Microbiology, University of Veterinary Medicine, 1210 Vienna, AustriaJoachim.spergser@vetmeduni.ac.at (J.S.); 2 Institute of Medical Microbiology and Hygiene, Austrian Agency for Health and Food Safety, 1090 Vienna, Austria; adriana.cabal-rosel@ages.at (A.C.R.); franz.allerberger@ages.at (F.A.); werner.ruppitsch@ages.at (W.R.); 3 Clinical Unit of Equine Surgery, University of Veterinary Medicine, 1210 Vienna, Austria; theresia.licka@vetmeduni.ac.at

**Keywords:** AmpC, ESBL, *Klebsiella pneumoniae*, antibiotic-resistance, β-lactamases, horses

## Abstract

**Simple Summary:**

Broad-spectrum cephalosporin-resistant *Klebsiella pneumoniae* is considered as a serious problem for public human health. To date, only a few broad-spectrum cephalosporin-resistant *Klebsiella* have been isolated from horses. Considering the zoonotic potential of the *Klebsiella* spp., and the close relationship between man and horse, this study intended to generate data on the genetic background of broad-spectrum cephalosporin-resistant *Klebsiella* spp. isolated from horses in Austria. Overall, samples isolated between 2012 and 2019 from 1541 horses underwent bacteriological testing, resulting in 51 specimens tested positive for *Klebsiella* ssp. Antimicrobial susceptibility tests revealed that seven *Klebsiella* ssp. isolates were not only cefotaxime-resistant but also showed resistance against other classes of antibiotics so that they were considered to be multidrug-resistant. Data from whole genome sequencing and mating experiments strongly suggest that the majority of antibiotic resistance genes is encoded on plasmids in these seven multidrug-resistant *Klebsiella* ssp. Considering the potential threat when commensal *Klebsiella* inhabiting a healthy human gut acquire new antibiotic resistances due to the exchange of plasmids with multidrug-resistant *Klebsiella* ssp. from horses, further monitoring of horses and other domestic animals for the presence of broad-spectrum cephalosporin-resistant *Klebsiella*, not only in Austria but worldwide is therefore advisable.

**Abstract:**

The aim of the present study was to investigate the diversity of broad-spectrum cephalosporin-resistant *Klebsiella* spp. isolated from horses in Austria that originated from diseased horses. A total of seven non-repetitive cefotaxime-resistant *Klebsiella* sp. isolates were obtained during diagnostic activities from autumn 2012 to October 2019. Antimicrobial susceptibility testing was performed. The isolates were genotyped by whole-genome sequencing (WGS). Four out of seven *Klebsiella* isolates were identified as *K. pneumoniae*, two as *K. michiganensis* and one as *K. oxytoca*. All isolates displayed a multi-drug resistant phenotype. The detection of resistance genes reflected well the phenotypic resistance profiles of the respective isolates. All but one isolate displayed the extended-spectrum β-lactamases (ESBL) phenotype and carried CTX-M cefotaximases, whereas one isolate displayed an ESBL and AmpC phenotype and carried cephamycinase (CMY)-2 and sulfhydryl variable (SHV)-type b and Temoniera (TEM) β-lactamases. Among *Klebsiella pneumoniae* isolates, for different sequence types (ST) could be detected (ST147, ST307, ST1228, and a new ST4848). Besides resistance genes, a variety of virulence genes, including genes coding for yersiniabactin were detected. Considering the high proximity between horses and humans, our results undoubtedly identified a public health issue. This deserves to be also monitored in the years to come.

## 1. Introduction

Among the member of the genus *Klebsiella*, broad-spectrum cephalosporin-resistant *Klebsiella* (*K.*) *pneumoniae* is frequently associated with severe nosocomial infections in humans, and due to its antibiotic-resistant traits, infections leave limited therapeutic options [1,2]. In early 2017, the World Health Organization (WHO) listed carbapenem-resistant and 3rd generation cephalosporin-resistant *Enterobacteriales* (including, e.g., *K. pneumoniae*, *Escherichia coli*, *Enterobacter* spp., *Serratia* spp., *Proteus* spp., *Providencia* spp., *Morganella* spp.) “Priority 1: Critical group” bacterial pathogens. These bacteria are in focus on the discovery and development of new antibiotics [2,3]. 

Today, broad-spectrum cephalosporin-resistant *K. pneumoniae* is recognized as a serious public health problem in human medicine [4,5]. Contrarily, there is still a scarcity of information on broad-spectrum cephalosporin-resistant *K. pneumoniae* and members of the genus *Klebsiella* isolated from horses and other domestic animals. To date, only a few equine broad-spectrum cephalosporin-resistant *Klebsiella* have been isolated and characterized [4,6,7,8,9,10]. Recent studies reported that some of the characterized resistant *K. pneumoniae* isolates of equine origin were human-associated multidrug-resistant (MDR) *K. pneumoniae* [4,8]. At present, there are no published data on the genetic background of broad-spectrum cephalosporin-resistant *Klebsiella* spp. isolated from horses in Austria. Therefore, there is a need to generate such data to understand the molecular epidemiology of these particular pathogens.

In the present study, we have characterized a collection of equine broad-spectrum cephalosporin-resistant *Klebsiella* sp. from clinical samples by multiphasic approach, including whole-genome sequencing (WGS).

## 2. Materials and Methods

At the Institute of Microbiology, University of Veterinary Medicine, Vienna, approximately 350 susceptibility tests are performed on clinical isolates from horses each year. During the study period (2012 until October 2019), samples of 1541 horses underwent bacteriological testing. In 51 specimens, *Klebsiella* sp. was detected, wherefrom a total of seven non-repetitive cefotaxime-resistant isolates, which were identified to the species level by matrix-assisted laser desorption/ionization-time-of-flight (MALDI-TOF) mass spectrometry (Bruker Daltonik, Heidelberg, Germany), and were further analyzed. They originated from lavage (isolates 1505 and 2826), wound (isolates 2668 and 2742), fistula (isolate 1635), trachea (isolate 2341b), and feces (isolate 4545). All isolates were stored in glycerol stocks at −80 °C. All samples originated from non-food producing horses. All these clinical samples were received from third parties and, therefore, not subject to reporting obligations of the Ethics and Animal Welfare Commission of the University of Veterinary Medicine in Vienna. 

Antimicrobial susceptibility testing was performed by agar disk-diffusion according to standards of the Clinical and Laboratory Standards Institute (CLSI) [11]. *Escherichia coli* ATCC^®^ 25922 served as quality control strains. The following antimicrobials were used: cefotaxime, ceftazidime, aztreonam, imipenem, meropenem, gentamicin, amikacin, tobramycin, ciprofloxacin, trimethoprim-sulfamethoxazole, tetracycline, chloramphenicol, and fosfomycin (Becton Dickinson, Heidelberg, Germany). In addition, isolates were checked for extended-spectrum β-lactamase (ESBL) production by ESBL-test via agar disk diffusion [11]. Furthermore, cefoxitin (30 μg) was added to this test to detect AmpC phenotypes.

Whole-genome sequencing (WGS) was performed by isolating and sequencing bacterial DNA, as previously described [12]. *De novo* assembly of raw reads, whole genome sequencingt (WGS) data analysis, including multi-locus sequence typing (MLST) and core genome multi-locus sequence-based typing (cgMLST), were performed, as previously described [13,14].

Species identification was conducted with the JSpecies workspace using the ANIb (average nucleotide identity via Basic Local Alignment Search Tool (BLAST) analysis tool [15]. The identification of acquired resistance genes and chromosomal mutations was performed using the Comprehensive Antibiotic Resistance Database (CARD; https://card.mcmaster.ca/home) [16], as well as ResFinder 3.2 (https://cge.cbs.dtu.dk/services/ResFinder/) [17] were used. eBURST (Based Upon Related Sequence Types) analysis (a plugin at https://bigsdb.pasteur.fr/) was conducted to identify clonal complexes (CCs), defined as groups of two or more independent isolates sharing identical alleles at six or more loci.

The presence of plasmids was determined using PlasmidFinder 1.3 available from the Center for Genomic Epidemiology web server (http://www.genomicepidemiology.org/) [18]. Probability Prediction of the location of a given antibiotic resistance gene was achieved by applying mlplasmids trained on *K. pneumonia* [19]. Posterior probability scores >0.7 and a minimum contig length of 1000 bp indicate that a given contig is plasmid-derived.

Mating experiments were conducted by conjugation as well as transformation, as previously described [20]. Variable regions of class 1 and class 2 integrons were determined by PCR [20]. The quinolone resistance-determining regions (QRDR) of *gyrA* and *parC* in ciprofloxacin-resistant isolates were amplified by PCR and sequenced [21].

The presence of virulence genes was examined by using the virulence allele library from the Institute Pasteur BIGSdb database for *K. pneumoniae* (http://bigsdb.pasteur.fr/klebsiella).

This whole-genome shotgun project has been deposited in DDBJ/EMBL/GenBank under the project number PRJNA600879. Raw sequence data for each strain were deposited under Sequence Read Archive (SRA) accession numbers SRR10899218 to SRR10899224.

## 3. Results

Four out of seven cefotaxime-resistant *Klebsiella* isolates were identified as *K. pneumoniae*, two as *K. michiganensis*, and one as *K. oxytoca* (Table 1). All but one isolate displayed the ESBL phenotype, whereas one isolate displayed an ESBL and AmpC phenotype. Besides cefotaxime, all *K. pneumoniae* isolates were resistant to ceftazidime, and one isolate additionally to aztreonam. All examined isolates were resistant to gentamicin and tobramycin. None of the analyzed isolates was resistant to carbapenems and amikacin. Five isolates were resistant to tetracycline, doxycycline, and chloramphenicol, whereas six were resistant to trimethoprim-sulfamethoxazole. All *K. pneumoniae* isolates were resistant to ciprofloxacin, and one isolate to fosfomycin (Table 1). Hence, all examined isolates were considered to be multidrug-resistant [22]. The detection of resistance genes reflected well the phenotypic resistance profiles of the examined isolates (Table 1). In two ciprofloxacin-resistant *K. pneumoniae* isolates, beside fluoroquinolone resistance genes *oqxA*, *oqxB*, *qrnB1*, and *aac(6′)-Ib-cr*, mutations in the quinolone resistance-determining regions (QRDRs) of the genes *gyrA* and *parC* were observed (Table 1). Three isolates, both *K. michiganensis* isolates and the *K. oxytoca* isolate contained a class 1 integron with a variable part of ca. 1.7 kb in size, which harbored an *aadA5* and a *dfr17* cassette.

In total, ten different plasmids IncFIA(HI1), IncFIB(K), IncFIB(pHCM2), IncHI1A, IncHI1B(R27), IncI1, IncN, IncQ1, IncR, and Col440l were identified (Table 2). They shared between 92.11 and 100% DNA similarity with corresponding reference sequences. A *K. michiganensis* isolate and two *K. pneumoniae* isolates carried IncFIA(HI1), IncFIB(pHCM2), IncHI1A, IncHI1B(R27), and IncQ1. The *K. oxytoca* isolate carried IncI1 and IncN, whereas a *K. pneumoniae* carried IncN and IncR and another *K. michiganensis* IncFIB(K). According to mlplasmids analyses, the majority of resistance genes might be located on plasmids, especially all *bla*_CTX_, *bla*_TEM_, and *bla*_OXA_ genes as well as all detected genes for resistance against aminoglycosides, trimethoprim/sulfamethoxazole, or chloramphenicol (Table 1).

Among virulence factors, *K. pneumoniae* type 3 fimbriae encoded by *mrk* operon genes as well *iutA* (aerobactin siderophores receptor) were detected in all *K. pneumoniae* isolates, whereas genes coding for yersiniabactin (*ybt*) were detected in only one isolate (Table 3). 

All four *K. pneumoniae* isolates belonged to different sequence types (ST) and cgMLST complex type (CT) (ST147-CT1202, ST307-CT4645, ST1228-CT4644 and a new ST4848-4643). These ST belonged to 4 clonal complexes: CC147 (ST147), CC37 (ST1228), CC307 (ST307) and CC702 (new ST4848). The minimum number of allelic differences between the isolates was 3686.

## 4. Discussion

The present study demonstrates that broad-spectrum cephalosporin-resistant members of the genus *Klebsiella* are present in the Austrian horse population, although the prevalence in clinical samples seems to be low. These findings are in accordance with previous studies describing the presence of these particular bacteria in horse populations of other countries [4,6,7,8,9,10]. Moreover, a previous study carried out in 2018 on clinical samples from Austrian patients reported 8.4% of *K. pneumoniae* isolates as resistant to third-generation cephalosporins [23].

In the present study, the most prevalent cefotaximase type was CTX-M-1 carried by all three *Klebsiella* species identified; this β-lactamase is commonly associated with *Enterobacteriales* from livestock [24]. CTX-M-15, the dominating cefotaximase, is considered the most common ESBL in *K. pneumoniae* from humans and animals worldwide [4]. To the best of our knowledge, the present study describes for the first time CTX-M-1 producing *K. michiganensis*, a close relative of *K. oxytoca*. One *K. pneumoniae* isolate that displayed both the AmpC and ESBL phenotype carried three different β-lactamases, *bla*_CMY2_, *bla*_SHV11_, and *bla*_TEM-1_. *bla*_CMY2_ was carried by an IncI1 conjugative plasmid. *K. pneumoniae* carrying plasmid-borne AmpC cephalosporinases (pAmpC) is a rare observation [6,25]. 

Another important observation is the co-existence of an arsenal of virulence factors and antibiotic resistance characters in one *K. pneumoniae* isolate (ST1228-CT4644). This isolate carried the yersiniabactin locus. Yersiniabactin is a siderophore, which is strongly associated with invasive clinical manifestations in humans [26]. Another siderophore, aerobactin, as well as type 3 fimbriae, which were detected in all *K. pneumoniae* isolates, may enhance colonization and adherence to host cells, invasiveness, and biofilm formation [27].

Among *K. pneumoniae* isolates examined, four different sequence types belonging to four different clonal complexes were identified. Two of these STs, ST147, and ST37, have been recognized as high-risk epidemic multiresistant human-associated clonal lineages [5]. ST147-CC147 is a human-related clone notorious for its multi-drug resistant character and harboring different β-lactamases, including carbapenemases [5]. Recently, this particular clone has emerged in companion animals [5,28]. In contrast, ST1228 has only one entry in the Institut Pasteur MLST database (http://bigsdb.pasteur.fr) and to our knowledge, had never been associated with horses. ST1228 belongs to CC37 whose predicted founder is ST37. *K. pneumoniae* ST37 isolates have been associated with different resistance properties, including carbapenem and colistin resistance, and were isolated from humans and animals [5,29]. One fecal isolate analyzed in the present study belonged to ST307-CC307. ST307 is a relatively new but highly successful pandemic clone, which was previously recovered from human patients, and recent data suggest a multi-drug resistant character of this clone [30]. β-lactamase producing *K. pneumoniae* ST307 has also been detected among different animals [31]. In the present study, a new sequence type, ST4848, belonging to the clonal complex CC702 (predicted founder ST702), has been identified by eBURST analysis. CC702 is a rare clone that has never been associated with broad-spectrum cephalosporin-resistant *K. pneumoniae* of equine origin. Data generated in this study (mating experiments, PlasmidFinder analysis, posterior probability plasmid analysis) strongly suggest that the majority of resistance genes are plasmid-borne. All identified replicons (IncFIA(HI1), IncFIB(K), IncFIB(pHCM2), IncHI1A, IncHI1B(R27), IncI1, IncN, IncQ1, IncR) are considered as vehicles of *bla*_CTX-M-15_ and *bla*_CTX-M-1_ dissemination in humans and animals [5,32]. 

## 5. Conclusions

Even though the overall prevalence of broad-spectrum cephalosporin-resistant *Klebsiella* sp. among specimens of equine origin in Austria appears to be low, the proportion of broad-spectrum cephalosporin-resistant *Klebsiella* spp. vs. non-resistant *Klebsiella* spp. is worth mentioning, since commensal *Klebsiella* spp. can acquire antimicrobial resistance. As such, the broad-spectrum cephalosporin-resistant *Klebsiella* spp. especially in combination with other resistance properties, are of special clinical importance because of dramatically narrowing the possibility of antibiotic treatment. Due to the regular contact and proximity between horses and humans monitoring horses for the presence of cephalosporin-resistant *Klebsiella* spp. is advisable in order to prevent further spread of these zoonotic agents.

## Figures and Tables

**Table 1:** Characteristics of seven examined cefotaxime-resistant *Klebsiella* isolates.

		1505		1635		2341b		2668		2742		2826		4545	
		*K. michiganensis*		*K. oxytoca*		*K. pneumoniae*		*K. pneumoniae*		*K. pneumoniae*		*K. michiganensis*		*K. pneumoniae*	
**ST ^1^**		n.a. ^2^		n.a.		ST4848		ST1228		ST147		n.a.		ST307	
**CT ^3^**		n.a.		n.a.		CT4643		CT4644		ST1202		n.a.		CT4645	
**Origin**		Lavage		Fistula		Trachea		Wound		Wound		Lavage		Feces	
			PPP ^4^		PPP		PPP		PPP		PPP		PPP		PPP
**β-lactamas**	P ^5^	CTX		CTX		CTX, CAZ, FOX		CTX, CAZ		CTX, CAZ, ATM		CTX		CTX, CAZ	
	G ^6^	***bla*_CTX-M-1_^7^**	0.745	*bla*_CTX-M-1_	0.710	***bla*_CMY2_**	*no ppp*	***bla*_CTX-M-1_**	0.760	*bla*_CTX-M-15_	0.973	*bla*_CTX-M-1_	0.753	*bla*_CTX-M-15_	0.976
		*bla*_OXY-4-1_	0.009	*bla*_OXY-2-7_	0.003	*bla*_SHV_	0.003	***bla*_SHV-11_**	0.001	*bla*_OXA-1_	0.961	*bla*_OXY-4-1_	0.004	*bla*_OXA-1_	0.961
				*bla*_TEM-1B_	0.891	*bla*_TEM-1B_	0.965	*bla*_TEM-1B_	*no ppp*	*bla*_SHV-11_	0.001			*bla*_SHV-28_	0.002
										*bla*_TEM-1B_	0.973			*bla*_TEM-1B_	0.776
**Aminoglycosides**	P	GEN, TOB		GEN, TOB		GEN, TOB		GEN, TOB		GEN, TOB		GEN, TOB		GEN, TOB	
	G	*aac(3)-IId*	0.854	*aac(3)-IId*	0.909	*aac(3)-IIa*	0.956	*aac(3)-IId*	0.92	*aac(3)-IIa*	0.963	*aac(3)-IId*	0.921	*aac(3)-IIa*	0.992
		***aadA5*^8^**	0.993	***aadA5***	0.955	*aph(3″)-Ib*	0.956			*aac(6′)-Ib-cr*	0.961	***aadA5***	0.986	*aac(6′)-Ib-cr*	0.961
		*aph(3″)-Ib*	0.896	*aph(3″)-Ib*	0.895					*aph(3″)-Ib*	0.973	*aph(3″)-Ib*	0.895	*aph(3″)-Ib*	0.976
		*aph(3′)-Ia*	*no ppp*	*aph(6)-Id*	0.895					*aph(6)-Id*	0.973	*aph(3′)-Ia*	*no ppp*	*aph(6)-Id*	0.976
		*aph(6)-Id*	0.896									*aph(6)-Id*	0.895		
**Tetracyclines**	P	TET, DOX		TET, DOX						TET, DOX		TET, DOX		TET, DOX	
	G	*tet*(B)	0.690	*tet*(B)	0.545					*tet*(A)	0.029	*tet*(B)	0.709	*tet*(A)	0.946
**Chloramphenicol**	P	CHL		CHL						CHL		CHL		CHL	
	G	*catA1*	0.993	*catA1*	0.945					*catB3*	0.961	*catA1*	0.986	*catB3*	0.961
**Trimethoprim/sulfamethoxazole**	P	SXT		SXT		SXT				SXT		SXT		SXT	
	G	*sul1*	0.993	*sul1*	0.955							*sul1*	0.986		
		*sul2*	0.896	*sul2*	0.895	*sul2*	0.956			*sul2*	0.973	*sul2*	0.895	*sul2*	0.976
		***dfrA17***	0.993	***dfrA17***	0.955	*dfrA14*	0.876			*dfrA14*	0.968	***dfrA17***	0.986	*dfrA14*	0.957
**Fosfomycin**	P							FOS		FOS				FOS	
	G							*fosA*	0.001	*fosA*	0.003			*fosA*	0.001
**Fluoroquinolones**	P					CIP		CIP		CIP				CIP	
	G					*oqxA*	0.002	*oqxA*	0.001	*oqxA*	0.001	*oqxA*	0.052	*oqxA*	0.001
						*oqxB*	0.002	*oqxB*	0.001	*oqxB*	0.001			*oqxB*	0.001
						*qrnS1*	0.631	*qnrS1*	0.760	*qnrB1*	0.029			*qnrB1*	0.946
										*aac(6′)-Ib-cr*	0.961			*aac(6′)-Ib-cr*	0.961
**QRDR ^9^**						wild type		wild type		*gyrA* (Ser83-Ile)				*gyrA* (Ser83-Ile)	
**QRDR**						Wild type		wild type		*parC* (Ser80-Ile)				*parC* (Ser80-Ile)	

^1^ ST—sequence type obtained after MLST; ^2^ n.a.—not applicable; ^3^ core genome multi-locus sequence-based type; ^4^ PPP—Posterior probability scores; ^5^ P—phenotypic resistance: CTX-cefotaxime, CAZ-ceftazidime, FOX-cefoxitin, ATM-aztreonam, GEN-gentamicin, TOB-tobramycin, TET-tetracycline, DOX-doxycycline, CHL-chloramphenicol, SXT-trimethoprim/sulfamethoxazole, CIP-ciprofloxacin; ^6^ G-genotypic resistance; ^7^ bold and underline—detected on transconjugats/transformants; ^8^ bold—within class 1 integron; ^9^ mutation in *gyrA* and *parC* of quinolone resistance-determining region (QRDR).

**Table 2:** Identified plasmids in *Klebsiella* isolates.

ID	Plasmid	Identity	Accession Number
1505	IncFIA(HI1)	100.0	AF250878
	IncFIB(pHCM2)	96.49	AL513384
	IncHI1A	99.52	AF250878
	IncHI1B(R27)	100.0	AF250878
	IncQ1	100.0	M28829.1
1635	IncFIA(HI1)	100	AF250878
	IncFIB(pHCM2)	96.49	AL513384
	IncHI1A	99.52	AF250878
	IncHI1B(R27)	100	AF250878
	IncQ1	100	M28829.1
2341b	IncI1	100	AP005147
	IncN	99.61	AY046276
2668	IncN	100	AY046276
	IncR	100	DQ449578
2742	Col440I	92.11	CP023920.1
2826	IncFIA(HI1)	100	AF250878
	IncFIB(pHCM2)	96.49	AL513384
	IncHI1A	99.52	AF250878
	IncHI1B(R27)	100	AF250878
	IncQ1	100	M28829
4545	IncFIB(K)	98.93	JN233704
	Col440I	94.74	CP023920.1

**Table 3:** Identified virulence factors in four *K. pneumoniae* isolates. Numbers correspond to the exact alleles detected.

Virulence Gene	2341b	2668	2742	4545	
*iutA*	new allele	new allele	new allele	new allele	aerobactin transport
*mrkA*	2	2	6	12	type 3 fimbrial gene cluster
*mrkB*	33	2	3	2	
*mrkC*	new allele	2	2	new allele	
*mrkD*	1	12	12	8	
*mrkF*	new allele	8	8	4	
*mrkH*	10	7	7	2	
*mrkI*	7	15	15	4	
*mrkJ*	19	12	12	2	
*ybtA*		1			yersiniabactin
*ybtE*		4			
*ybtP*		4			
*ybtQ*		22			
*ybtS*		6			
*ybtT*		1			
*ybtU*		14			
*ybtX*		15			
*fyuA*		17			
*irp1*		44			
*irp2*		37			
